# Cannabinoids and Viral Infections

**DOI:** 10.3390/ph3061873

**Published:** 2010-06-09

**Authors:** Carol Shoshkes Reiss

**Affiliations:** Department of Biology, Center for Neural Science, NYU Cancer Institute and Department of Microbiology, New York University, 100 Washington Square East, New York, NY, 10003, USA; E-Mail: carol.reiss@nyu.edu; Tel.: +1-212-998-8269; Fax: +1-212-995-4015

**Keywords:** pathogens, virus infection, immunomodulation, inflammation

## Abstract

Exogenous cannabinoids or receptor antagonists may influence many cellular and systemic host responses. The anti-inflammatory activity of cannabinoids may compromise host inflammatory responses to acute viral infections, but may be beneficial in persistent infections. In neurons, where innate antiviral/pro-resolution responses include the activation of NOS-1, inhibition of Ca^2+^ activity by cannabinoids, increased viral replication and disease. This review examines the effect(s) of cannabinoids and their antagonists in viral infections.

## 1. Introduction

Both endogenous and exogenous cannabinoids can influence the course of infections *in vitro* and *in vivo*. This review will focus on viral infections of mammals, but will also describe what is known about other infections. Readers are directed to the excellent accompanying reviews in this issue which expertly discuss the clinical trials, cell biology, mechanisms of action, impact on inflammation, clinical applications, and so forth.

Cannabinoids may act either through the CB_1_ or the CB_2_ receptor, which are found on distinct cell types. The CB_1_ receptor is found on neurons as well as some astrocytes and skeletal muscle cells; neurons are frequently the target of viral infection. Engagement of the CB_1_ receptor by its endogenous or exogenous agonists may inhibit the release of Ca^2+^ from intracellular or extracellular stores. Since many important intracellular proteins are Ca^2+^-dependent for activation, signal transduction through the CB_1_ receptor may impair these secondary pathways and have a profound influence on the ability of viruses to replicate in neurons.

In contrast, the response of cells expressing the CB_2_ receptor may influence not only the responses in that cell, but may alter the course of the host innate and adaptive immune response to the pathogen, suppressing inflammation and the development of virus-specific cellular and humoral responses. The outcome on the viral infection will depend on whether inflammation is beneficial or pathogenic in the specific case.

## 2. Discussion

When a host is infected with a virus, there is a dynamic competition between the ability of the host to first marshal innate (hours to days) and then adaptive immunity (>7 days post infection) *vs.* the replication and spread of the virus first within the host and then to additional susceptible individuals. When a virus is able to out-pace the containment efforts, the host may succumb. Pathology may result from damage to tissues by viral-induced cellular apoptosis or necrosis, or alternatively, host immune responses may result in immunopathology or the perceived symptoms of the infection. If, however, innate and adaptive immunity successfully suppress viral replication, specific life-long immunity may result.

In order to understand the influences on the host response which may be the result of cannabinoids, it is important to examine some of the cellular pathways which are dependent on Ca^2+^-dependent enzymes. Table 1 indicates some of the well characterized pathways involved and their potential impact on viral infections.

The common recurring impact of Ca^2+^-dependent enzymes is a role in inflammation. This ranges from regulation of many signal transduction pathways, production of pro-inflammatory and pro-resolving lipid mediators downstream of arachidonic acid, to activation of Nitric Oxide Synthase and the production of reactive nitrogen intermediates, to proteolytic enzymes which remodel the cytoskeleton or extracellular matrix, and apoptosis.

Inflammation is essential for recruitment of both innate and adaptive immune cells to the site of infection to control virus production and limit spread, and then to promote recovery. Inflammation is comprised not only of non-specific cells (sequentially these are polymorphonuclear leukocytes, natural killer cells, macrophages) and then pathogen-specific T lymphocytes recruited from circulation, and activation of antibody-secreting B lymphocytes, but also induction of production and secretion of cytokines, chemokines, interferons, complement components, acute phase reactants, reactive oxygen and nitrogen intermediates, and other mediators [24,25,26]. Readers are referred to the accompanying review by Bani, Mannaioni, Passani, and Masini [27]. Thus, many of these critical pathways may be impaired or compromised when endogenous or exogenous cannabinoids are present during an infection [28]. 

Cannabinoids have been used both recreationally by groups of people who have viral infections, and experimentally by scientists investigating their impact *in vitro* or in animal models. Table 2 presents what has been published about these populations in peer reviewed journals. In most of the infections studied (Table 2), it is apparent that cannabinoid treatment, whether *in vitro* or *in vivo*, had profound impact on the virus-host (cell) interactions. For HSV-2, HIV-1, KSHV, influenza and VSV viral replication, or surrogate measures of infection, were found to be substantially increased upon cannabinoid treatment [30,34,39,50,52,63]. In HIV-1 infection, syncytia formation was enhanced, and monocytes were stickier on endothelial cells [57,58]. In one study, KHSV was more likely to exit latency and enter lytic infection when transformed cells were treated with THC [39], however, another study found the opposite result in several herpesvirus infections [38].

**Table 1:** Some Ca^2+^-dependent enzymes which may be inhibited by Cannabinoids and speculated role in host responses relevant for viral infections.

Enzyme primary/secondary	Pathways	Ref.	Role(s) in viral infection-host responses
cPhospholipase A_2_	Arachidonic acid metabolites (prostaglandins, leukotrienes, lipoxins, resolvins) and inflammation	[1,2]	Inflammation and its resolution
Phospholipase C - Receptor-mediated tyrosine kinase	Production of Inositol 1,4,5-triphosphate from phosophotidylinositol	[3]	Signal transduction
Phospholipase D_1_	Exocytosis in neuroendocrine cells	[4]	Neurotransmission
Calcineurin	Activation of NFAT—gene expression	[5,6]	Signal transduction
Ca^2+^-Calmodulin - Nitric oxide synthase-1 - Nitric oxide synthase-3	Conversion of argenine to NO in neurons and endothelial cells; production of ONOO-, -SNO, -R-NO_2_ Inhibition of viral infection	[7,8,9,10,11,12]	Anti-viral; NO_2_-decoration of viral proteins; capillary dilation; inflammation
Ca^2+^-Calmodulin dependent protein kinases - CREB - CaMKK activation of AMPK	Wnt-2-dependent dendrite growth & cardiomyogenesis Energy, epithelial cell polarity T cell activation	[13,14,15,16,17]	Adaptive immune responses; inflammation
Calpains [Ca^2+^-dependent proteases]	Neutral proteases [many tissues] Cell membrane fusion, synaptic remodeling, activating PKC, remodeling cytoskeleton, transcription factors	[18,19,20]	Cytoskeletal plasticity, cell migration, inflammation
Matrix metalloproteinases	Extracellular matrix remodeling, inflammation	[21]	Inflammation
Calpastatin	Cell fusion in fertilization	[22]	Formation of heterokaryons /giant cells
Transglutaminases	Cross-linking/deamination of proteins –wound healing, tissue repair, apoptosis, cell cycle control, inflammation and fibrosis	[23]	Inflammation, fibrosis, cell cycle and programmed cell death

**Table 2:** Cannabinoids and Viral Infections.

Viral pathogen	*In vivo In vitro*	Agonist / Antagonist	Titer change	Pathogenesis	Inflammation Immunoregu-lation	Comments	Ref.
HSV-2, *L. monocyto-genes*	*In vivo*	Δ9-THC		decreased resistance to LD_50_		systemic infection	[29]
HSV-2	*In vivo*	Δ9-THC	increased shedding	increased severity of lesions & mortality	delayed onset of DTH response	vaginal model B6C3H F_1_ mouse	[30]
HSV-2	*In vivo*	Δ9-THC			decreased Type I IFN response	i.v. infection	[31]
HSV-2	*In vivo*	Δ9-THC		decreased resistance to infection; increased severity of lesions		vaginal guinea pig model	[32]
HSV-1,-2	*In vitro*	Δ9-THC	failed to replicate			antiviral effect in human & monkey cells	[33]
HSV-2	*In vitro*	Δ9-THC	100-fold increase in released virus			Vero cells, increased CPE	[34]
HSV-2	both	Δ9-THC			decreased T cell proliferation	B6C3H F_1_ mice immunized then T cells cultured	[35]
HSV	*In vitro*	Δ9-THC	decreased infectivity in TC			virus incubated with THC	[36]
HSV-1	both	Δ9-THC			decreased CD8 CTL activity	C3H mice immunized, L929 targets	[37]
EBV, KSHV, HVS, HSV-1, MHV-68	*In vivo*	Δ9-THC	Immediate early ORF promoter activity inhibited	reactivation from latency inhibited		latently infected B cells in tissue culture	[38]
KSHV	*In vivo*	Δ9-THC	increased viral load	increased efficiency of infection, activation of lytic switch	increased transformation of endothelial cells	primary human dermal microvascular cells	[39]
Cowpox	*In vivo*	Marijuana cigarettes		generalized infection	weak Ab production, no neutralizing Abs	Case report	[40]
TMEV	*In vitro*	Anandamide			decreased release of NO2- and TNF-α	NO is antiviral for TMEV	[41,42]
TMEV	*In vitro*	Anandamide			increased IL-6 production	astrocyte culture B6 and SJL mice	[43]
TMEV	*In vivo*	WIN-55,212		ameliorates progression of autoimmune disease TMEV-IDD	decreased DTH, decreased IL-1, IL-6, IFN-γ , TNF-α,	TMEV-IDD a mouse model of MS	[44]
TMEV	*In vivo*	OMDM1, OMDM2		ameliorated motor symptoms	decreased MHC II, inhibited NOS-2, reduced proinflammatory cytokines	TMEV-IDD proposed MS therapy with cannabinoids	[45]
TMEV	*In vitro*	JWH-133 SR144558		role of CB_2_ receptors in anti-inflammatory actions	reduced IL-12p40, reduced ERK1/2 signaling		[46]
TMEV	*In vitro*	WIN-55,212		CB_2_-dependent COX-2 induction increased vs. TMEV-alone	role of PI3 kinase pathway in CB_2_ but MAPK for TMEV signaling	proposed role on blood-flow and immune activity	[47]
TMEV	*In vivo*	Palmitoyl-ethanol-amine		reduction in motor disability in TMEV-IDD	anti-inflammatory effect	TMEV-IDD	[48]
TMEV	both	WIN-55,212		inhibited ICAM & VCAM on endothelium; role for PPAR-γ receptors in mechanism	reduced inflammation	TMEV-IDD	[49]
Influenza	*In vivo*	Δ9-THC	HA mRNA increased	inflammation, metaplasia of mucous cell	decreased CD4, CD8, and macrophage recruitment		[50]
Influenza	*In vivo*	Δ9-THC	HA mRNA decreased in CB_1_/CB_2_KO mice	THC-mediated airway pathology +/- CB_1_/CB_2_	KO mice had increased CD4 and IFN-γ recruitment	CB_1_/CB_2 _KO mice	[51]
VSV	*In vitro*	WIN-55,212	increased viral titers	CB_1_-dependent; decreased NOS-1 activity	antagonized IFN-γ-mediated antiviral pathway	suggested disease progression likely in neurons/viral encephalitis	[52]
BDV	*In vivo*	WIN-55,212		protected BrdU-positive neural progenitor cells in striatum	suppressed microglial activation	suggested treatment of encephalitis with microglial inflammation and neuro-degeneration	[53]
HCV	*In vivo*	Marijuana cigarettes		progression of liver fibrosis		epidemiological study	[54]
HCV	*In vivo*	Oral cannabinoids		improved weight	no viral markers or immune markers studied	7 week clinical trial for anorexia and nausea	[55]
HCV	*In vivo*	Marijuana cigarettes		progression of liver fibrosis; increased disease severity		clinical pathological survey of 204 HCV patients	[56]
HIV-1	*In vitro*	Δ9-THC, CP-55,940, WIN-55,212		increased syn-cytia formation MT-2 cells (CB_1_ & CB_2_^+^)		speculate cannabinoids enhance HIV-1 infection	[57]
HIV-1	*In vitro*	anandamide		increased adherence for monocytes	uncoupled NO release, inhibited NO	human saphenous vein or internal thoracic artery; speculate higher titers *in vivo*	[58]
HIV-1 Tat	*In vitro*	WIN-55,212		reduced tat-induced cytotoxicity	inhibited NOS-2 activity	C6 rat glioma cell line	[59]
HIV-1	*In vivo*	Marijuana cigarettes		increased appetite	insufficient numbers of individuals	3 week trial	[60]
HIV-1	*In vivo*	Marijuana cigarettes	mRNA unchanged		CD4+ and CD8+ cells unchanged	3 week trial, placebo-controlled	[61]
HIV-1		WIN-55,212	inhibited expression			CD4 and microglial cultures	[62]
HIV-1	*In vivo*	THC	increased viral replica-tion 50-fold		decreased CD4 IFN-γ-producing cells, increased co-receptor expression	scid-Hu mouse model	[63]
HIV-1 Gp120	*In vitro*	2-AG, CP55940		inhibited Ca^+2^-flux-induced substance P, decreased permeability		model of BBB, co-culture of Human brain microvascular endothelial cells and astrocytes	[64]
HIV-1	*In vivo*	WIN-55,212		dose-related hypothermia in mouse pre-optic anterior hypothalamus infusion	WIN-55,212 is antagonist for SDF-1a/ CXCL12/ CXCR4 [HIV-1 coReceptor] pathway	mouse model for HIV-thermoreg-ulation by direct injection of WIN-55,212 to brain POAH center	[65]
HIV-1 Tat	*In vitro*	CP55940, Δ9-THC		CB_2_-dependent inhibition of U937 migration to Tat	possible anti-inflammatory mechanism	U937 cells in culture	[66]

Legend: BDV, Borna disease virus; EBV, Epstein-Barr virus; HCV, Hepatitis C virus; HIV, Human immunodeficiency virus; HSV, Herpes simplex virus; HVS, Herpes virus samirii; KO, knock-out mice; KSHV, Kaposi's sarcoma herpes virus; *L. monocytogenes, Listeria monocytogenes*; MHV-68, Murine herpes virus-68; TMEV, Theiler's murine encephalomyelitis virus; VSV, Vesicular stomatitis virus.

Disease was more severe in HSV-2-infected guinea pigs which were treated with THC [29,30,32]. In HCV infections, clinical studies have shown a profound co-morbidity of recreational cannabinoid use, for disease progression [54,56]. One case report of Cowpox infection, a very rare human pathogen, indicated that recreational use of cannabinoids was associated with generalized infection and very poor immune responses to the virus [40].

In contrast, in those infections where host inflammatory responses are often associated with pathology, and not with clearance and recovery, cannabinoid treatment of hosts was beneficial. These included one mouse model of multiple sclerosis, the Theiler's murine encephalomyelocarditis virus (TMEV)-induced demyelinating disease (IDD), where progression towards the paralysis and disability were ameliorated [44,45,48] and in Borna disease virus (BDV) where neural progenitors were protected from proinflammatory cytokine-mediated damage [53] infections. TMEV-IDD is characterized by microglial activation in the spinal cord of mice and a T cell-mediated autoimmune demyelinating disease, triggered by the viral infection [42,67,68,69]. Persistent BDV infection of the central nervous system is associated with immunopathology associate with inflammation and production of pro-inflammatory cytokines, induction of NOS-2 in microglia, and breakdown of the blood-brain barrier [70,71,72,73]. In both BVD and TMEV-IDD, the targets for the anti-inflammatory effects of the cannabinoid treatment are lymphocytes and mononuclear cells.

Two excellent reviews of the impact of cannabinoids on bacterial, yeast, and protozoan infections were published in the same issue of *Journal of Neuroimmunology* [26,74]. These infections included *Treponema pallidum* (Syphilis), *Legonella pneumophila* (Legionnaires' disease), *Staphylococci aureus* and* S. albus*, *Listeria monocytogenes, Candida albicans* (Thrush), and *Naegleria fowleri*. Both reviews concluded that THC significantly reduced host resistance to infection of experimental animals, and speculated that similar host compromise would be found in man. In the more than 12 years since those reviews were published, additional findings have extended the serious consequences of cannabinoids on host responses to pathogens and opportunistic infections. Marijuana use is a risk factor for *Mycobacterium tuberculosis* (TB) infections [75,76,77]; this author speculates the suppression of host innate immune responses by THC contributes to the increased severity of TB in users. Similarly, more serious exacerbations central nervous system infection by *Acanthamoeba* among HIV-infected patients has been attributed to marijuana consumption [78], possibly by inhibiting macrophage chemotaxis [79]. However, the antiinflammatory effects of cannabinoids have been found to be beneficial in attenuating fever induced by bacterial endotoxin [65,80], inhibiting cytokine responses to *Corynebacterium parvum* endotoxin [81]. These drugs may also offer therapeutic efficacy in meningitis caused by *Streptococcus pneumoniae* [82] and in irritable bowel syndrome [83,84].

Cannabinoids may relieve pain and may induce hyperphagia, which could be beneficial in cancer [85,86]. However, these physiological characteristics are not relevant to most viral, bacterial fungal or parasitic infections, where the regulation of inflammation is central to controlling pathogen replication and immunopathology. However, the same anti-inflammatory properties of cannabinoids just described are detrimental to the host in handling the other infections. In most cases, a rapid and robust inflammatory response, associated with production of proinflammatory cytokines and effect T lymphocytes capable of eliminating infected cells is essential to recovery and survival.

## 3. Conclusions

Cannabinoids are profoundly anti-inflammatory and impair many Ca^2+^-dependent enzyme systems which are central to inflammatory and cell-autonomous antiviral responses. When viral-induced host responses lead to immunopathology, as is seen in a rodent model of multiple sclerosis, TMEV-IDD, or in a persistent infection of the central nervous system caused by a non-lytic virus, BDV, cannabinoid treatment was beneficial.

In all other virus infections, both *in vitro* and *in vivo*, cannabinoid treatment led to disease progression, increased pathology, and sometimes to host death. Therefore, in many clinical settings, including latent infections caused by HIV-1 or HSV-1, and persistent infection of the liver caused by HCV, cannabinoids lead to worsened disease outcome.
